# Rigors

**Published:** 1899-12-09

**Authors:** 


### RIGORS.

Rigors are apt to occur from such a multitude of causes, and they take place in the course of such a variety of diseases, that however significant they may be as warnings of evil they cannot be said to be very important as guiding symptoms in the diagnosis of disease. Occurring, as they do, at the commencement of many maladies, they are in such cases interesting, principally as marking the date of onset, and as to some extent giving a hint as to the severity of the attack, but they say little as to the nature of the on-coming disease. When, however, they occur in the course of an illness they give most important indications, showing pretty positively that something has happened—some change, some new departure, the intercurrence of some fresh complication, or the implantation of some new infection; and, perhaps, it is as an indication of the occurrence of some such disturbance in the natural course of a malady, and as marking the date of the fresh complication, that rigors are of the greatest interest, and have their most important diagnostic significance. In a clinical lecture delivered a short time ago at St. Bartholomew's Hospital Sir Dyce Duckworth described the great variety of conditions in which rigors might be met with. But first for his picture of the condition itself. The tremors, he says, involve the whole body and the limbs, and often cause chattering of the teeth. The face is pinched and the expression is distressed. The skin is pallid and dry, and the fingers are shrunken; the lips and nails are dusky; the feet, hands, nose, and ears may be cold to the touch; the respiration is hurried and shallow; the pulse is frequent and wiry—that is, small—and of high tension; the tongue is moist; and the temperature of the body is raised, although the patient has the sensation of extreme cold, especially over the back and round the abdomen. The mind remains clear. Now such a condition as this occurring in the course of an illness is nearly always a matter of considerable importance; it sounds a note of alarm, and suggests the possibility of suppuration or of some toxic infection. If, for example, in the course of an otitis media, of endocarditis, or of hepatitis with jaundice, a rigor occurs, and is repeated at irregular intervals, we suspect the occurrence of cerebral abscess, or infective endocarditis (arterial pyæmia), or hepatic abscess, or possibly suppurative portal phlebitis. Then, again, the occurrence of a rigor in a puerperal patient is one of the terrors of the accoucheur, suggesting, as it does, a fear of uterine sepsis or puerperal fever. Such rigors occurring in the puerperium may also indicate the onset of parametritis or perimetritis, and in connection with abortion or retained placenta may indicate septic intoxication or sapræmia. In connection with a venous thrombosis of the limbs or elsewhere one or more rigors may occur. Then the onset of acute tuberculosis is sometimes attended with repeated paroxysms of rigors, fever, and sweats, which may closely simulate aguish attacks. In tuberculous kidneys and calculous pyelitis the same may be witnessed as a result of septic infection. Occasionally, also, rigors take place in profound anæmia, in Hodgkin's disease, and in rapidly-growing cancer. In the course of fevers such attacks not infrequently depend on fresh infection by some other toxin than that proper to the original malady; the severe rigors occurring late in the course of typhoid fever are probably thus to be explained. We do not see much of surgical pyæmia nowadays, but the occurrence of rigors after operations in times gone by used to be the almost unerring sign of the onset of some such blood infection. The relation between rigors and infection by pus-producing organisms must always be remembered, for we are thus led to note the important fact that the occurrence of a rigor in a medical case often at once indicates the necessity for surgical interference.

